# Validity of the Mental Health Continuum – Short Form among home-language Setswana speaking South Africans: evidence for a four-factor model

**DOI:** 10.3389/fpsyg.2025.1547673

**Published:** 2025-05-21

**Authors:** Ingrid Opperman, Johan C. Potgieter, Jessica Daniel-Smit

**Affiliations:** ^1^Optentia Research Unit, North-West University, Vanderbijlpark, South Africa; ^2^COMPRES Research Unit, North-West University, Potchefstroom, South Africa

**Keywords:** psychometric validity, bifactorial model, confirmatory factor analysis, Mental Health Continuum – Short Form (MHC-SF), Setswana home language

## Abstract

From a positive psychology perspective, it has been proposed that mental health comprises three dimensions: emotional well-being (EWB), psychological [or personal] well-being (PWB), and social well-being (SWB). To assess these dimensions, Keyes (2002) developed the Mental Health Continuum – Short Form (MHC-SF), which has been validated in various cultural contexts. In this model, mental health is operationalized as the presence of various positive indicators rather than the absence of psychopathology in a model which is purported to be cross-culturally applicable. While numerous studies support the original, correlated three-factor model, some current arguments are being made for a bifactor model with three dimensions. However, few newer validation studies explore the possibility of alternate models which might be applicable to non-Western, collectivist cultures who can also benefit from accurate assessments and positive psychology interventions. This study assessed the validity of the MHC-SF among 308 Setswana home-language South Africans aged 19–31 years. Results indicated that the correlated three-factor structure or bifactor model validated previously was a good fit, but a correlated four-factor model was a better fit and a bifactor model with four dimensions was the best fitting. An initial exploratory factor analysis using maximum likelihood and promax rotation suggested that this was due to the social well-being scale being divisible into: “belonging in society” (social 1) and “perceptions of society” (social 2) rather than a single construct (social well-being). These results emphasize the distinct aspects of well-being in a Setswana-speaking sample, highlighting the importance of culturally and linguistically informed models of mental health, particularly in collectivistic cultures within developing countries. This has implications for the use of the MHC-SF in research and culturally appropriate assessment and intervention design, as well as the applicability of fundamental models of mental health in non-Western and African contexts.

## 1 Introduction

From a positive psychology perspective, mental health is defined as a state of well-being, wherein individuals can realize their full potential, effectively cope with normal life stresses, and actively contribute to their community (Afrashteh and Janjani, 2023; Galderisi et al., 2015; Keyes, 2002; World Health Organization [WHO], 2022). This perspective underscores the importance of three integral components for mental health: well-being, optimal functioning at a personal level, and optimal functioning within a community context (Khazaei et al., 2023). Additionally, it emphasizes increasing recognition of the fact that mental health is not necessarily the absence of mental illness but rather the presence of a sense of positive, subjective well-being (Galderisi et al., 2015; Keyes, 2002). Keyes (2002) posited three dimensions of mental health: emotional well-being related to life satisfaction and feelings of happiness; psychological/personal well-being related to self-realization and positive functioning; and social well-being characterized by positive social relationships and societal significance. To assess mental health composite of these dimensions, Keyes (2002) developed the Mental Health Continuum – Short Form (MHC-SF), a measure of general mental health or well-being encompassing the three dimensions.

The MHC-SF offers a valuable tool for assessing mental health across diverse populations, which has led to continues adaptation and/or translation of the instrument as well as numerous validation studies focused on evaluating the psychometric properties. Psychometric properties of the English version of the MHC-SF have been extensively studied across diverse populations, including French-Canadian population (Doré et al., 2017); Indonesian population (Faradiba et al., 2023); Iranian population (Khazaei et al., 2023); Hungarian population (Reinhardt et al., 2020); New Zealand population (Pir et al., 2021); Spanish population (Echeverría et al., 2017); Serbian population (Joshanloo and Jovanović, 2017); and South African population (de Bruin and du Plessis, 2015; Keyes et al., 2008).

Confirmatory factor analyses have supported a clear three-factor model in various studies (de Bruin and du Plessis, 2015; Doré et al., 2017; Keyes et al., 2008; Lamers et al., 2011; Petrillo et al., 2015) while others have additionally proposed a bifactor model with three dimensions as a better fit (de Bruin and du Plessis, 2015; Hides et al., 2016; Jovanović, 2015; Longo et al., 2020; Monteiro et al., 2020; Shannon et al., 2023; Söderqvist and Larm, 2021; Yeo and Suárez, 2022). Confirmatory factor analyses conducted by Longo et al. (2020) showed that the bifactor model fit was superior to the three-factor model in Polish adults, Dutch adolescents and adults, Portuguese children, and Serbian adults where RMSEA values for the three-factor model were consistently poorer in all samples. In another Serbian population, the bifactor model fit was superior for both adolescents (RMSEA = 0.060) and adults (RMSEA = 0.047) (Jovanović, 2015). Additionally, RMSEA values of 0.53 or better were obtained across several French-Canadian samples for a bifactor model with three dimensions (Lamborn et al., 2018). In post-partum Portuguese women, Monteiro et al. (2020) reported RMSEA values of 0.076 for the correlated three-factor model and 0.064 for a bifactor model, where an ECV value of 0.82 suggested considerable unidimensionality in this particular case. Hides et al. (2016) reported similar values for an Australian sample (RMSEA = 0.06 for a bifactor model with three dimensions), also finding substantial unidimensionality (*ω*_h_ = 0.905), as did Söderqvist and Larm (2021) for Swedish adolescents (RMSEA = 0.061, *ω*_h_ = 0.79, ECV = 0.73) and Yeo and Suárez (2022) in a Singaporean (RMSEA = 0.08, *ω*_h_ = 0.92, ECV = 0.85) and an Australian (RMSEA = 0.05, *ω*_h_ = 0.89, ECV = 0.76) sample. Conversely, de Bruin and du Plessis (2015) reported similar fit statistics for a South African sample, but less unidimensionality (RMSEA = 0.06, ECV = 0.63).

Monteiro et al. (2020; Portuguese sample) reported factor loadings of mostly >0.700 for all items in a correlated three factor model, and the relative strength remained stable in bifactor circumstances. However, Petrillo et al. (2015; Italian sample), de Bruin and du Plessis (2015; South African sample) and Lamers et al. (2011; Dutch sample) reported moderate item-factor loadings of > ~ 0.50 for most items on the scale. Notably, items four and five on the social well-being scale consistently demonstrated considerably poorer loadings across all studies. Additionally, these studies showed lower unidimensionality and loadings to the general factor were similar to loadings to the three dimensions. In more unidimensional data, Hides et al. (2016; Australian sample) also reported cross-loading of item 4 in all models and factor loadings of <0.40 in contrast to the other item loadings which ranged from 0.57 to 0.92 while Söderqvist and Larm (2021; Swedish adolescent sample) reported generally strong factor loadings of >0.5 in most cases to the general factor, but weak loadings to the three dimensions in a bifactor model. As for other research in other samples, items four and five had notably poor loadings for the traditional three factors.

Although studies have found strong support for the original, correlated three-factor model (e.g., Doré et al., 2017; Keyes et al., 2008; Pir et al., 2021; Tejada-Gallardo et al., 2024), differential findings concerning the item construction of the model are still prevalent. Some research has suggested poor discriminability between the three factors despite good model fit indices, suggesting a unidimensional model where substantial proportions of common variance were explained by a general factor (Hides et al., 2016; Longo et al., 2020; Rogoza et al., 2018; Söderqvist and Larm, 2021; van Erp Taalman Kip and Hutschemaekers, 2018; Yeo and Suárez, 2022). Thus, current thinking tends toward a bifactor model with three broad dimensions, but predominantly a general well-being factor. However, other studies have not suggested excessive unidimensionality but have noted that interrelationships of the items within the factors, particularly for the social well-being scale, differ somewhat from the hypothesized model (de Bruin and du Plessis, 2015; Joshanloo, 2020; Lamborn et al., 2018; Lamers et al., 2011). It is unknown whether differences are due to changes in understandings of mental health in terms of item structure, newer techniques of investigation such as bifactor modeling and exploratory structural equation modeling, or a function of differing translations among language groups where the MHC-SF was administered in English to non-native speakers. Particularly in South Africa, little recent work has considered the structure of the MHC-SF in relation to specific language groupings (cf. de Bruin and du Plessis, 2015 [South African students]; Keyes et al., 2008 [Setswana adults]) although a few studies have noted that metric and partial scalar invariance do exist between South African and other samples (Joshanloo et al., 2013). Therefore, conceptualizations of mental health among the distinct language groups in the South African population may be more unique than previously anticipated. This article focuses on assessing the three-factor model of the MHC-SF in a sample of South African adults with Setswana as a home language.

## 2 Materials and methods

### 2.1 Participants

Data from 308 Setswana home language, young adults (19–31 years, *M* = 24.47 ± 3.22 years) were obtained from the African-PREDICT study. The sample were 54.9% (*n* = 169) female and 45.1% (*n* = 139) male. The majority were never married (*n* = 281; 91.2%) and had a high school (*n* = 177; 57.5%) or tertiary (*n* = 105; 34.1%) education. Most of the sample were employed (*n* = 169, 54.9%) or self-employed (*n* = 5; 1.6%) with the remainder being unemployed (*n* = 134; 43.5%) who were looking for work (*n* = 65; 21.1%) or students (*n* = 62; 20.1%) or did not provide a response (*n* = 174; 56.5%). Household incomes were in the low socio-economic status bracket, primarily <R9,999 per month (*n* = 246; 79.9%). Approximately half of the sample were receiving social grants of some form (*n* = 152; 49.4%) with no missing data.

### 2.2 Instruments and procedures

#### 2.2.1 Mental Health Continuum – Short Form

The Mental Health Continuum – Short Form (MHC-SF) examines the emotional, psychological, and social well-being components of mental health from a positive psychology perspective, and places a person on a continuum ranging from flourishing (presence of a high level of mental health) to languishing (indicating its relative absence) (Keyes and Simoes, 2012; Keyes, 2002). The MHC-SF comprises 14 items (3 for emotional well-being, 5 for social well-being, and 6 for psychological [personal] well-being) measured on a 6-point Likert-type scale (0 = “never” to 5 = “every day”) of how frequently they felt a certain way. Scores can range from 0 to 70 for the total scale, 0 to 15 for the emotional well-being scale, 0 to 25 for the social well-being scale, and 0 to 30 for the psychological (personal) well-being scale. Higher scores suggest higher levels of positive well-being or mental health.

The emotional well-being scale (EWB) focuses on happiness [item 1: “positive emotions (E)”], interest in life [item 2: “interest (E)”], and life satisfaction [item 3: “satisfied (E)”]. The social well-being scale (SWB) focuses on contributions to society [item 4 (S): “social contribution”], belonging in the community [item 5: “social integration (S)”], whether society is becoming a better place for people like the participant [item 6: “social actualization (S)”], whether the participant believes people are basically good [item 7: “social acceptance (S)”], and whether the way society works makes sense [item 8: “social coherence (S)”]. The psychological well-being scale (PWB) focuses on how frequently the participant considers how much they like about their personality [item 9: “self-acceptance (P)”], whether the participant feels as though they are good at managing responsibilities in daily life [item 10: “environmental mastery (P)”], warm and trusting relationships with others [item 11: “positive relations (P)”], experiences which have challenged growth [item 12: “personal growth (P)”], confidence in expressing ideas and opinions [item 13: “autonomy (P)”], and whether the participant feels that they have a sense of direction and meaning in life [item 14: “purpose in life (P)”].

#### 2.2.2 Procedure

Data on Setswana home-language speakers were sub-sampled from the African-PREDICT study, a longitudinal initiative focusing on predictors and correlates of markers in the development of cardiovascular disease, coupled with psychosocial measurements to enhance prevention programs. Recruitment in the African-PREDICT study was voluntary via advertisements and invitation via health screening clinics and fieldworkers. At the time of assessment, participants in were healthy, had not been previously diagnosed with chronic illnesses or HIV, did not have blood pressure readings exceeding the normal threshold, and were not pregnant or breastfeeding in the case of female participants. All participants were literate in written and spoken English.

Participants in the African-PREDICT study completed the MHC-SF as part of a battery of demographic questions, psychosocial questionnaires, physical health measurements (e.g., cardiovascular), and biological sampling. The psychological questionnaires were administered hard copy (paper/pencil) by professionally trained psychological practitioners who were available for questions. Data were captured electronically by trained personnel. The sub-sample of Setswana home language speakers was obtained based on the demographic information provided. No exclusion criteria were put in place other than completion of the MHC-SF.

### 2.3 Statistical analysis

Descriptive statistics on the average MHC-SF scores were calculated for the total sample and subgroups for gender, marital status, education level, employment status, and grant status. Confirmatory factor analyses (CFA) using maximum likelihood estimation, suitable for normal and non-normal data with lower risk of sampling bias (Gerbing and Anderson, 1985; Li, 2016) were conducted in *R*’s *Lavaan* package (Rosseel, 2012) for the known models: a unidimensional model (Hides et al., 2016; Santini et al., 2020), the correlated three-factor model (Kennes et al., 2020; Keyes et al., 2008; Lamers et al., 2011; Luijten et al., 2019), as well as an additional model based on an exploratory factor analysis by the maximum likelihood method with promax rotation to allow for the expected correlated (oblique) factors representing general mental health (G). The Kaiser criterion of eigenvalues greater than or equal to 1.00 was used for extraction (Braeken and van Assen, 2017; Hayton et al., 2004; Tabachnick et al., 2018). Cronbach’s *α* and McDonald’s *ω*_t_ were used to assess internal consistency of the total scale and subscales of the correlated models.

Exploratory bi-factor analyses were conducted using the Schmid–Leiman transformation as the orthogonalization method to allow for free loading of the factors to a general factor (mental health) and domain-specific factors (Jung et al., 2020), namely, the theoretically-derived three dimensions (see de Bruin and du Plessis, 2015; Joshanloo and Jovanović, 2017; Lamborn et al., 2018; Rogoza et al., 2018; Söderqvist and Larm, 2021) and the model identified in the exploratory factor analysis. McDonald’s ω*_h_* and the ECV were used to assess the proportion of variance due to a single construct and the extent of unidimensionality.

The Root Mean Square Error of Approximation (RMSEA) and Standardized Root Mean Square Residual (SRMR) were used for absolute fit indices comparing the built model’s fit to the data and the Tucker-Lewis Index (TLI) was used to assess incremental fit of the model to a null model. Threshold values of RMSEA ≤ 0.06, SRMR ≤ 0.08, and TLI ≥ 0.95 indicated a good fit. Common variance of a general factor (unidimensionality) was assessed using the explained common variance (ECV) and McDonald’s ω*_h_* (Rodriguez et al., 2015).

## 3 Results

### 3.1 Descriptive statistics

The sample had a mean score of 48.65 (*SD* = 10.75) for total well-being, 11.16 (*SD* = 2.92) for EWB, 13.34 (*SD* = 5.59) for SWB, and 24.15 (*SD* = 4.96) for PWB (Table 1).

**Table 1:** Descriptive statistics for the mental Health Continuum – Short Form (*n* = 308).

Dimension	Mean	Standard deviation	Possible range
Emotional well-being	11.16	2.92	0–15
Social well-being	13.34	5.59	0–25
Psychological well-being	24.15	4.96	0–30
Total well-being	48.65	10.75	0–70

### 3.2 Exploratory factor analysis

An exploratory factor analysis using maximum likelihood and promax rotation revealed four factors based on extraction of eigenvalues greater than 1.00. Barlett’s test of sphericity was statistically significant (*χ*^2^ = 1307.05, *p* = 0.001) and the Kaiser–Meyer–Olkin measure of sampling adequacy was 0.848 indicating factorability of the data. Extraction sum of squares loadings explained a total of 49.080% of variance with the four rotated sum of squares loadings explaining 3.349% of variance, 3.250% of variance, 2.294% of variance, and 1.962% of variance over the four extracted factors (Table 2).

**Table 2:** Exploratory factor analysis using maximum likelihood extraction and promax rotation.

Factor	Initial eigenvalues			Extraction sums of squared loadings			Rotation sums of squared loadings^a^
	Total	% of variance	Cumulative %	Total	% variance	Cumulative %	Total
1	4.743	33.879	33.879	4.158	29.702	29.702	3.349
2	1.624	11.602	45.482	1.096	7.828	37.53	3.25
3	1.196	8.544	54.025	0.895	6.396	43.926	2.294
4	1.153	8.239	62.265	0.722	5.154	49.08	1.962
5	0.744	5.315	67.579				
6	0.684	4.888	72.467				
7	0.627	4.48	76.946				
8	0.608	4.344	81.29				
9	0.538	3.84	85.13				
10	0.506	3.613	88.743				
11	0.463	3.31	92.053				
12	0.416	2.97	95.023				
13	0.364	2.601	97.623				
14	0.333	2.377	100				

^a^When factors are correlated, sums of squared loadings cannot be added to obtain a total variance.

The four-factor solution produced a split in the SWB scale (social 1 and social 2). Items 6 [“better society (S)”], 7 [“people good (S)”], and 8 [“society works (S)”] loaded to social 1 while items 4 [“societal contribution (S)”] and 5 [“belonging (S)”] loaded to social 2. Some cross-loadings between factors 1 (PWB) and 2 (EWB) were present for items 10 [“responsibilities (P)”] and 11 [“relationships (P)”]. Excepting item 11, loadings were larger than 0.400 with the majority larger than 0.600 (Table 3).

**Table 3:** Pattern matrix.

Item	Psychological	Emotional	Social
Happy (1E)		0.709	
Interest (2E)		0.502	
Satisfied (3E)		0.878	
Societal contribution (4S)			0.684
Belonging (5S)			0.747
Better society (6S)		0.465	
People good (7S)		0.472	
Society works (8S)		0.948	
Personality (9P)	0.530		
Responsibilities (10P)	0.407	0.312	
Relationships (11P)	0.336	0.295	
Growth (12P)	0.812		
Opinions (13P)	0.722		
Meaning (14P)	0.637		

Coefficients <0.250 have been suppressed for clarity.

### 3.3 Confirmatory factor analysis

#### 3.3.1 Unidimensional model

**Figure 1:**
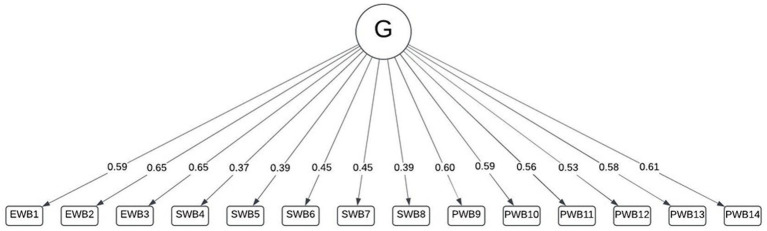
Univariate model of general well-being.

Initially, model fit for a unidimensional model was calculated under the assumption that the MHC-SF measures a general construct. The unidimensional model had a poor fit (RMSEA = 0.121; SRMR = 0.090, TLI = 0.670). The loadings indicate that variance in the items associated with the social well-being scale (items 4–8) was particularly poorly explained, but the scale did have strong internal consistency (*α* = 0.835; *ω*_t_ = 0.825) (Figure 1).

#### 3.3.2 Three-factor models

**Figure 2:**
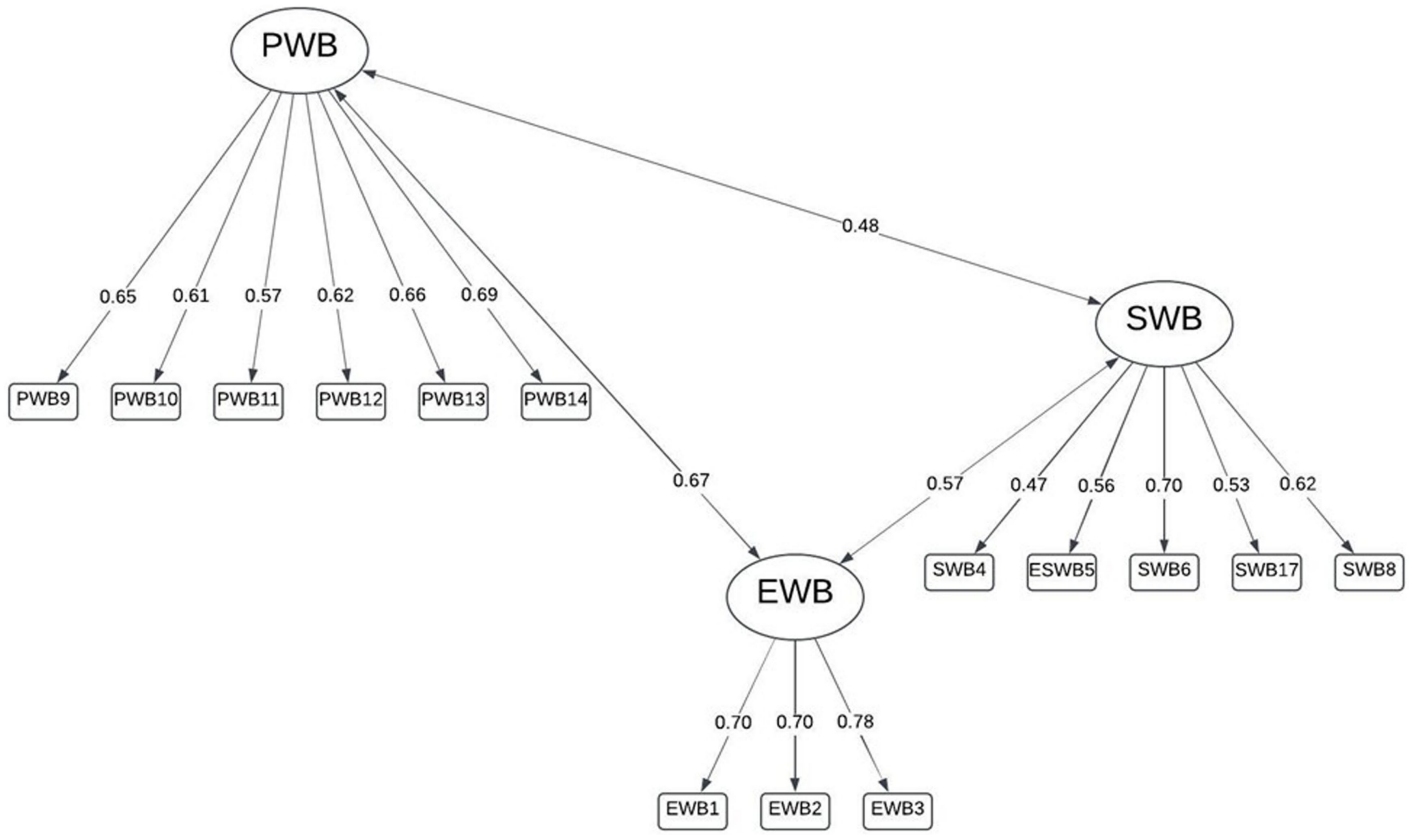
Three-factor model identified by Keyes (2002).

Maximum likelihood estimation of the theoretically defined, correlated three-factor model resulted in a moderate fit (RMSEA = 0.078, SRMR = 0.060, TLI = 0.863). Covariances between the latent factors suggested shared variance between the PWB and EWB scales as suggested in the pattern matrix from the exploratory factor analysis with reference to items 10 and 11. As reported in other research, the internal consistency of the three subscales was relatively strong (EWB: *α* = 0.762; *ω*_t_ = 0.774; SWB: *α* = 0.718; *ω*_t_ = 0.699; PWB: *α* = 0.798; *ω*_t_ = 0.798) (Figure 2).

**Figure 3:**
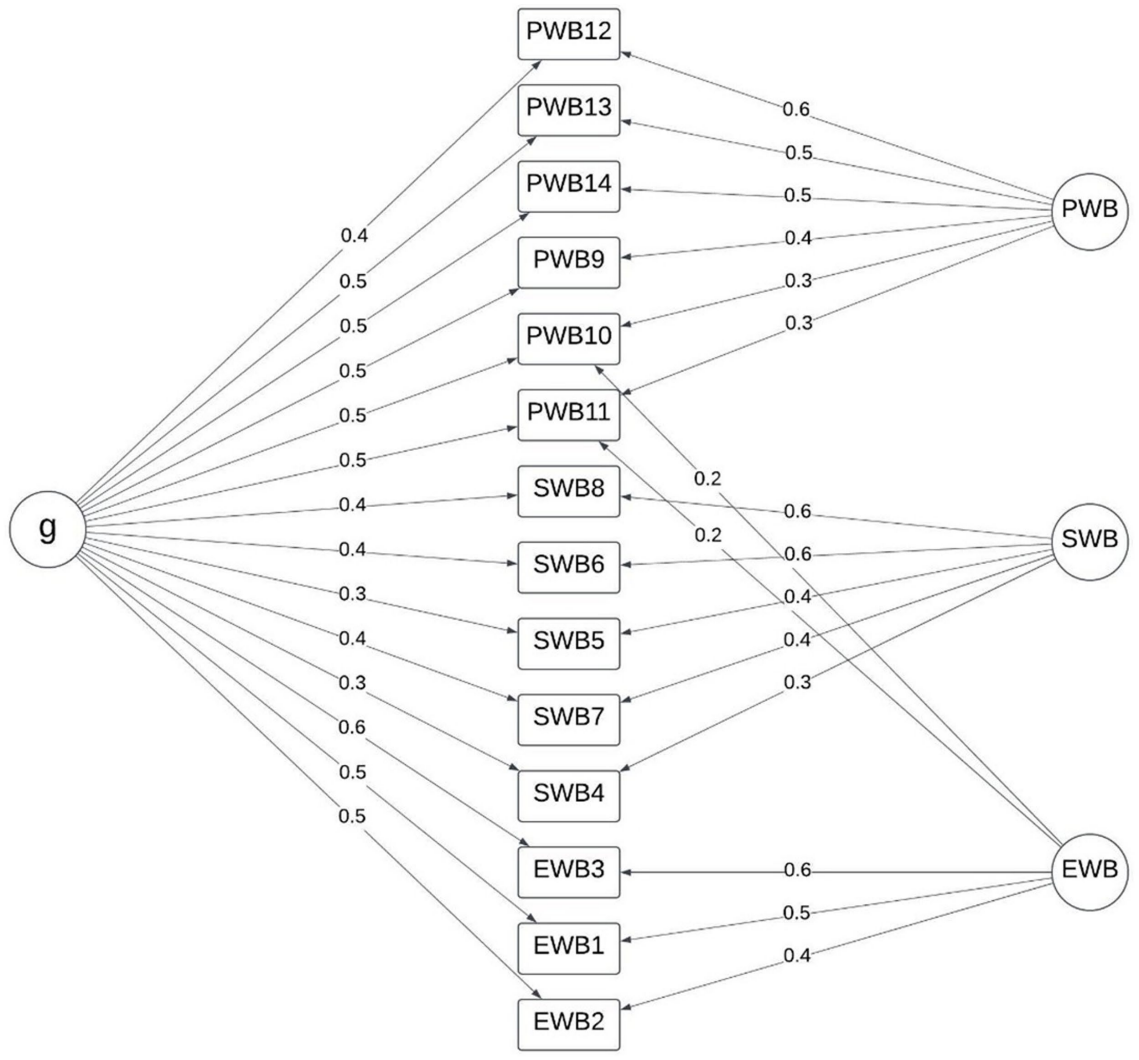
Bifactor model with Keyes (2002) three dimensions.

A bifactor model for the hypothesized three dimensions specified orthogonal rotation (uncorrelated factors) to allow for free loading given the presence of a general well-being dimension being included. The bifactor model had a similar fit when three factors were specified which was still superior to the single, general factor model (RMSEA = 0.075, SRMR = 0.060). The explained common variance was relatively low (ECV = 0.48), suggesting multidimensionality, as was omega hierarchical (ω*_h_* = 0.600). Loadings to the general factor were moderate, but cross-loadings of items 10 (“responsibilities”) and 11 (“relationships”) between the EWB and PWB subscales were present (Figure 3).

#### 3.3.3 Four-factor model

**Figure 4:**
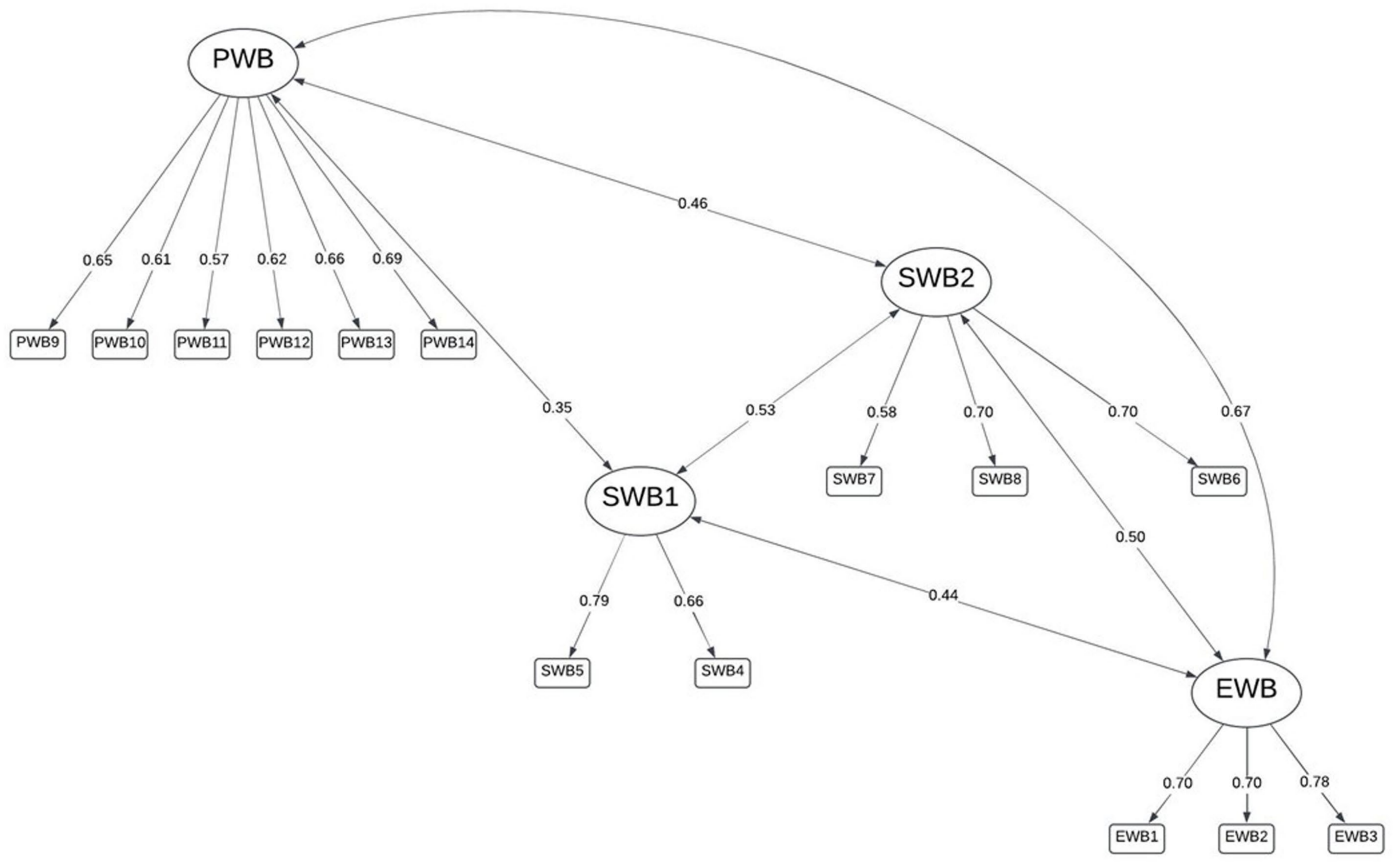
Four-factor model: emotional well-being, psychological [personal] well-being, social 1 (belonging in society), and social 2 (perceptions of society).

Maximum likelihood estimation of the four-factor model derived from the exploratory factor analysis resulted in a good to moderate fit (RMSEA = 0.061, SRMR = 0.051, TLI = 0.915). Dimensions for social 1 and social 2 had similar shared variance with PWB as that in the three-factor model, as did EWB with PWB. Similarly, shared variance between the social 1, social 2, and EWB was lower than that between the EWB and SWB scales. The shared variance between social 1 and social 2 was not notable, suggesting that they are independent latent constructs and distinctly separable from the PWB and EWB scales which are more strongly associated (Figure 4).

**Figure 5:**
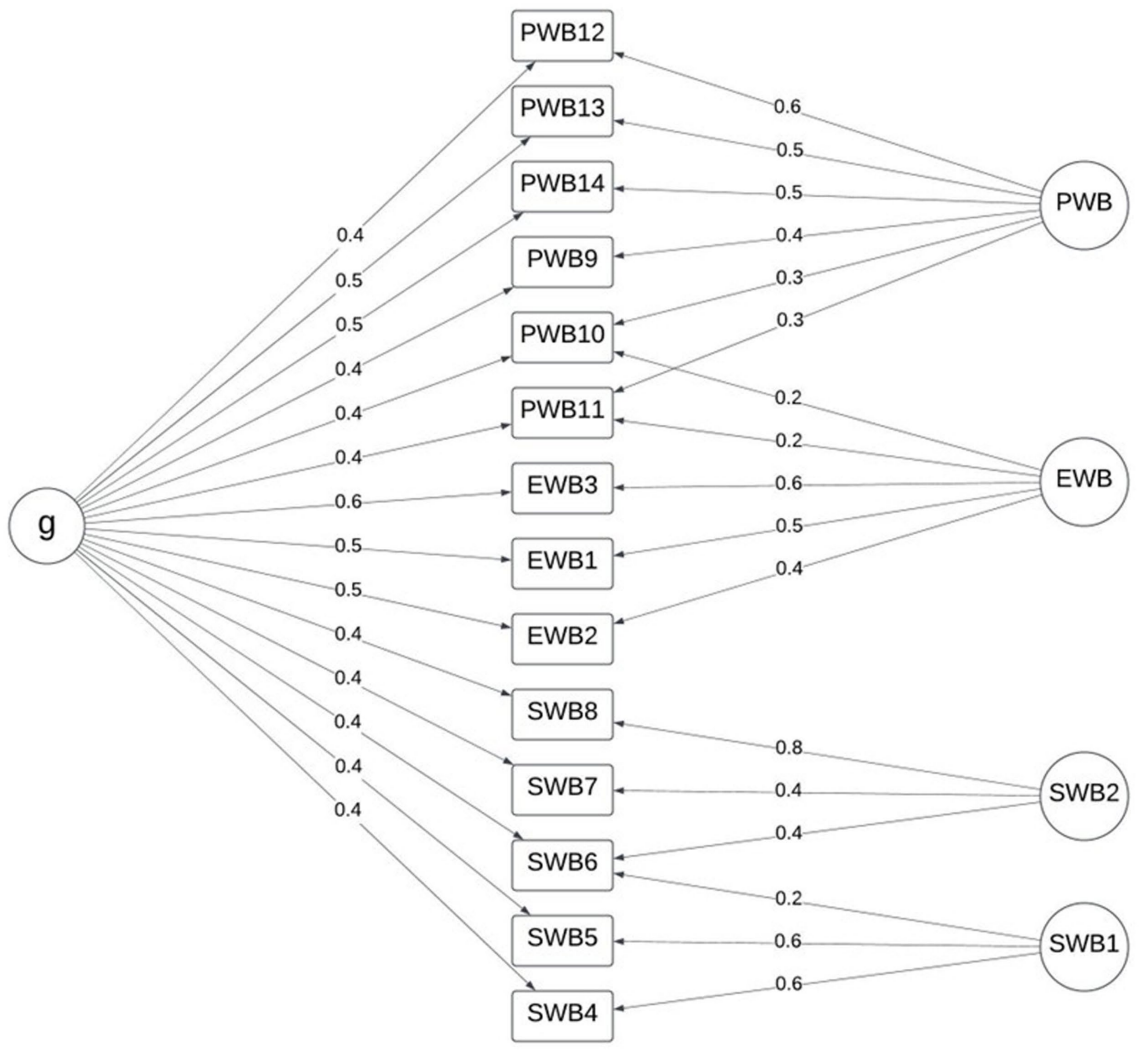
Bifactor model with four dimensions.

A bifactor model using the four dimensions was best fitting (RMSEA = 0.034, RMSR = 0.020) and the explained common variance did not suggest unidimensionality (ECV = 0.42, ω*_h_* = 0.600), but did confirm the presence of a general well-being factor, as hypothesized. The bifactor model with three dimensions, as in the other models, cross-loadings were evidenced for items 10 [“responsibilities (P)”] and 11 [“relationships (P)”] between the PWB and EWB factors (see also Table 3); however, excepting a cross-loading for item 6 [“better society (S)”], not clearly evidenced in the exploratory factor analysis pattern matrix (Table 3), the separated social dimensions were clearly defined with similar loading values to the psychological and emotional dimensions’ items (Figure 5).

The data indicate that although a general well-being factor is present, the MHC-SF exhibits a bifactor structure with four dimensions in Setswana speakers rather than three. In this model, less than half of the ECV is accounted for by a general well-being construct and the model fit is substantially superior to a three-factor model or bifactor model with three dimensions which have been suggested in some research (e.g., de Bruin and du Plessis, 2015; Hides et al., 2016; Joshanloo and Jovanović, 2017; Jovanović, 2015; Lamborn et al., 2018), which is also inferior to a four-factor model. Table 4 summarizes the model fit indices for the models tested. It is noteworthy that while the RMSEA and RMSR values improved for the four-dimension bifactor model, the TLI declines fractionally. The data suggest clear dimensionality in both cases.

**Table 4:** Summary of model fit indices.

Model	RMSEA	TLI	RMSR	ECV	ω*_h_*
Unidimensional	0.121	0.670	0.090		
3-factor	0.078	0.863	0.060		
Bifactor (3)	0.075	0.863	0.060	0.48	0.6
4-factor	0.061	0.915	0.051		
Bifactor (4)	0.034	0.908	0.020	0.42	0.6

## 4 Discussion

The correlated four-factor structure identified in the exploratory factor analysis (maximum likelihood, promax rotation) based on Kaiser’s criterion had a superior fit to the correlated three-factor structure or bifactor model with three dimensions, despite the latter structures having been validated previously (cf. de Bruin and du Plessis, 2015; Keyes et al., 2008). Although multiple other studies have found a correlated three-factor model or bifactor model with three dimensions suitable, data has often suggested poorer loading of items 4 (“societal contribution”) and 5 (“belonging”) on the SWB scale. The present study’s exploratory and confirmatory factor analyses suggest that these two items may be separable in a distinct social well-being scale rather than combined with items 6, 7, and 8 in the original three-factor structure even if bifactorial solutions with three dimensions are considered (c.f. de Bruin and du Plessis, 2015). Furthermore, the covariances suggested that the four factors were interrelated, but not uniform, and explained common variance and omega hierarchical did not suggest unidimensionality of the instrument. Therefore, for this sample of South African home-language Setswana speakers, a correlated four-factor model or bifactor model with four dimensions are superior to three-factor structures without unidimensionality.

Explanations of mental health from which the MHC-SF was derived considered a general dimension of well-being, as reflected in the uncorrelated bifactor models in this sample, comprising a combination of subjective positive affect and life satisfaction (hedonic well-being) and positive functioning (eudaimonic well-being) (Keyes, 2002). In this sample, positive affect and life satisfaction as it represents a state of ‘feeling good’ or emotional/hedonic well-being (Schutte and Wissing, 2017) was well delineated in the data. On the other hand, aspects of functioning well or eudaimonic well-being from a psychological perspective (Schutte and Wissing, 2017) appeared to overlap to a certain extent, as evidenced in the cross-loadings for items 10 (managing “responsibilities” of daily life) and 11 (warm, trusting “relationships” with others), both of which could be conceptualized from both a ‘feeling good’, or ‘functioning well’ perspective. This may align with other studies suggesting high levels of unidimensionality in the MHC-SF (e.g., Hides et al., 2016; Söderqvist and Larm, 2021; Yeo and Suárez, 2022) with an overriding general well-being factor with a negative–positive well-being continuum.

Positive functioning (eudaimonic) and feeling good (hedonic) aspects of well-being are both influenced by positive relationships (Bailey and Miller, 1998; Manzi et al., 2006; Szcześniak and Tułecka, 2020; Vandeleur et al., 2009). The concept of positive affect (EWB) is established in the Setswana-speaking South African context (Wissing et al., 2010), but functional PWB may manifest differently due to a variety of other factors, such as personality (Schmutte and Ryff, 1997), interpersonal, contextual-demographics (Joshanloo et al., 2019; Khumalo et al., 2012), and personal versus social aspects (Joshanloo, 2021). Similar findings have been evidenced in variable factor loadings among non-English first language speaking populations, although a formal deviation from the three-factor model of the MHC-SF has not been debated. Nonetheless, it is possible that these factors may operationalize differently in the separate home-language groupings assessed making the model worth further investigation in non-English home language speakers.

The SWB scale on the MHC-SF focuses on belonging in society [item 5: “social integration (S)”], contributions to society [item 4 (S): “social contribution”], the betterment of people in society [item 6: “social actualization (S)”], which are perceived as generally good [item 7: “social acceptance (S)”], and whether society works as a whole [item 8: “social coherence (S)”]. In line with the empirical data, these can be viewed as encompassing two perspectives: (1) the person within society; and (2) the person’s view of society. The person “in” society encompasses whether the individual believes that they can contribute to how society functions and that they belong within a societal structure (social 1, items 4 and 5). The person’s view “of” society concerns the betterment of society in general, whether people are viewed as good, and whether society works (social 2, items 6, 7, and 8). This separation may speak to viewing the self as interdependent within society, linking social factor 1 to higher well-being, versus objectively evaluating the effectiveness of how society functions. In collectivistic cultures, such as the Setswana grouping, personal happiness is often intertwined with a sense of belonging in society (Krys et al., 2019; Lykes and Kemmelmeier, 2014) which is not necessarily related to a positive perception of how society functions or whether the people in society can be positively viewed. Alternatively, more individualistic mindsets come to the fore in items 1, 4, and 5 while more collectivistic value systems which promote non-criticism of society and harmonization are represented by items 2, 6, and 7.

A separable sense of subjective, affective well-being and subjective, functional well-being could attribute to the additional social dimension observed in the current data when a collectivistic, contextual mindset in which the individual is seated is considered. In this regard, other research has rather suggested a combination of the EWB and SWB scales in countries such as Kenya and Iran, both of whom are representative of collectivistic cultures (Żemojtel-Piotrowska et al., 2018).

In this study, the data suggests that in the collectivistic Setswana culture, societal contributions and belonging in society represent distinct factors which are still part of well-being. Making use of two social factors rather than one allows for a split between belonging in society and perceptions of society, which may be important in collectivistic cultures, particularly in developing countries. The subtle split between the elements of SWB may inform interventions aimed at facilitating the overall well-being of individuals in the Setswana and other African cultural groups. According to the Positive activity model developed by Lyubomirsky and Layous (2013), the efficacy of so-called positive psychology interventions (PPI’s) depends largely on the degree of person-activity fit, with an optimal fit between features of the person and the activity being strongly predictive of positive well-being or mental health outcomes. This implies that not all people benefit from positive psychology-type activities and interventions in the same way. As a result, a nuanced approach to the facilitation of positive psychology concepts, particularly social well-being in non-Western cultural contexts, is required.

## 5 Conclusion

The present study was conducted on a group of home-language Setswana speakers fluent in English who completed the MHC-SF as part of a larger battery of psychological tests. Limited support was found for the known three-factor model of the MHC-SF, with better fit indices for a bifactor three-dimensional model which is congruent with recent literature. However, exploratory factor analysis identified four factors, notably a separation in the SWB between the concepts of belonging in society and perceptions of society. This four-factor model fit was further improved as a bifactor model with four dimensions where unidimensionality was not suggested. The apparent distinction between belonging in society and perceptions of society may be reflective of the person interdependent with society in terms of belonging, and PWB as part of a collectivistic culture. The cross-loadings observed between some PWB items and EWB items were also reflective of societally-related concepts such as relationships. Alternatively, a distinction between individuality and harmonious societies may result in a similar cross-loading reflective of the Western-African distinction. These deserve further investigation in future research.

Although these findings are of interest in conceptualizations of the structure of the MHC-SF beyond the assumption of three or fewer factors, several limitations exist. The sample of Setswana home-language speakers is relatively homogeneous and from a small area. Most participants had undergone secondary schooling in a Western-based system, and the diversity of the schools attended is unknown. Nonetheless, Western-based schooling systems may contribute to shifts in societal perceptions, promoting a more individualistic view which can be contradictory to more traditional African values. Therefore, links between collectivistic cultures and conceptions of societal well-being are tentative and require clearer sampling in future investigations. Level of home language and English proficiency was not accounted for in this study, only fluency. Since the participants had completed secondary schooling, sufficient English fluency was likely, but translational errors for some terms in the items were not accounted for. Lastly, the sample were drawn from a semi-urban area without accounting for urban versus rural community living, the specifics of which may be associated with differing levels and points of mental health in general, particularly in low socio-economic status cases (Khumalo et al., 2012). Perceptions of society in low socio-economic areas of developing countries may differ substantially from the ideal society, which may also influence the distinction between belonging in a community as being separable from broader society and how it functions. Despite these limitations, the details of the social well-being scale in homogeneous language groups in South Africa deserve further investigation and may provide insight into young South Africans perceptions of well-being as members of society separate to the functioning of society.

## Data Availability

The datasets presented in this article are not readily available because data are proprietary and confidential held by the original research project coordinators, scientific committees, and institution. Dissemination of any data, including anonymized data, requires formal permissions. Requests to access the datasets should be directed to ingrid.opperman@nwu.ac.za.
